# Twice as Effective? Pressurized Intra-Thoracic Aerosol Chemotherapy: New Frontiers in Pleural Mesothelioma

**DOI:** 10.3390/medsci13020072

**Published:** 2025-06-02

**Authors:** Maria Giovanna Mastromarino, Elena Guerrini, Raffaele Guerrieri, Gianmarco Elia, Alessandra Lenzini, Vittorio Aprile, Greta Alì, Stylianos Korasidis, Marcello Carlo Ambrogi, Marco Lucchi

**Affiliations:** 1 Division of Thoracic Surgery, Cardiac, Thoracic and Vascular Department, University Hospital of Pisa, 56124 Pisa, Italy; alessandralenzini2@gmail.com (A.L.); aprilevittorio@gmail.com (V.A.); stylianoskorasidis@gmail.com (S.K.); marcello.ambrogi@unipi.it (M.C.A.); marco.lucchi@unipi.it (M.L.); 2 Department of Surgical, Medical and Molecular Pathology and Critical Care Medicine, University of Pisa, 56124 Pisa, Italy; elena.guerrini.pi@gmail.com (E.G.); raffaeleguerrieri@yahoo.it (R.G.); gianmarco.elia.7@gmail.com (G.E.); 3 Unit of Pathological Anatomy, Department of Surgical, Medical and Molecular Pathology and Critical Care Medicine, University Hospital of Pisa, 56124 Pisa, Italy; greta.ali@unipi.it

**Keywords:** pressurized intra-thoracic aerosol chemotherapy (PITAC), mesothelioma, malignant pleural effusion, local antineoplastic control, palliative care, pleural mesothelioma

## Abstract

Pressurized intra-thoracic aerosol chemotherapy (PITAC) is a novel and promising strategy for the treatment of malignant pleural effusion (MPE). PITAC enables effective pleurodesis while potentially exerting an antineoplastic effect by delivering chemotherapeutic agents as a therapeutic aerosol into the thoracic cavity via a nebulizer. Our preliminary study involved nine patients with unresectable pleural mesothelioma (PM) treated with PITAC. Among them, one case was particularly emblematic for demonstrating notable oncological improvements in addition to well-known palliative benefits. This patient underwent two PITAC procedures, one year apart, without perioperative complications. Redo pleural biopsies from both previous and new sites revealed only fibrous tissue and inflammatory cells, with no evidence of malignancy. Beyond achieving pleurodesis, PITAC—by combining cytotoxic and sclerosing effects—may offer effective local antineoplastic control and represent a promising avenue for enhancing loco-regional therapy in PM.

## 1. Introduction

Malignant pleural effusions (MPEs) occur in up to 15% of cancer patients and are associated with a poor prognosis due to pleural carcinomatosis [1]. Survival is generally limited, with a median ranging from 3 to 12 months, depending on the patient’s clinical comorbidities and the biological characteristics of the primary tumor [1]. In such cases, treatment remains primarily palliative, aiming to relieve symptoms and slow their progression [2].

Among the causes of MPE, malignant mesothelioma (MM), a rare cancer primarily linked to asbestos exposure, typically originates in the pleura (73–85%) and less commonly in the peritoneum (7–18%) [3,4,5,6].

Pleural mesothelioma (PM) predominantly affects males (with a male-to-female ratio of 5:1), and the risk increases with age, with a higher prevalence observed among individuals over 65 years of age [7,8]. A latency period of approximately 30 years generally elapses between the initial asbestos exposure and PM diagnosis. The absence of a reliable screening method to detect the disease early contributes to diagnostic delays [9,10]. Survival ranges from 12 to 30 months in localized disease and from 8 to 14 months in advanced stages [11,12]. Most PM cases are diagnosed at an advanced stage, and first-line therapy extends survival by an average of only three months [13]. The most common clinical manifestation is progressive dyspnea, usually secondary to MPE, with or without non-pleuritic chest pain resulting from chest wall invasion.

For resectable diseases, PM treatment is based on a trimodal approach: surgery, chemotherapy (neoadjuvant and/or adjuvant, including hyperthermic intrathoracic chemotherapy—HITHOC), and radiotherapy, especially in patients without lymph node involvement [14,15]. However, the prognosis remains poor, as only a small fraction of PM cases are considered resectable. Furthermore, most patients are inoperable at diagnosis due to advanced disease, comorbidities, or poor performance status.

In the large group of patients with unresectable disease, systemic chemotherapy remains the standard treatment. Platinum-based regimens demonstrate higher response rates compared to platinum-free options in the first-line setting [16]. Recently, the CheckMate-743 trial, a randomized phase III study, compared dual immunotherapy (ipilimumab and nivolumab) with platinum-based chemotherapy (cisplatin/carboplatin plus pemetrexed) as a first-line treatment for unresectable PM, establishing immunotherapy as the new standard for metastatic or unresectable non-epithelioid PM [17,18].

Due to the lack of curative options, palliative care plays a critical role in PM management. One of the most debilitating symptoms is MPE, which leads to progressive dyspnea, as previously noted. The ERS/ESTS/EACTS/ESTRO guidelines recommend talc poudrage via thoracoscopy as the first-line strategy for pleurodesis in patients with expandable lungs. For those unresponsive to chemical pleurodesis or indwelling catheters, palliative video-assisted thoracoscopic surgery (VATS) partial pleurectomy is advised for symptom control [19].

In recent years, intrapleural administration of chemotherapeutic agents has been explored to combine antineoplastic effects with pleurodesis [20,21,22]. In this context, pressurized intra-thoracic aerosol chemotherapy (PITAC) has emerged as a novel and promising therapeutic approach [23,24]. Adapted from abdominal surgery, where it has demonstrated efficacy in end-stage peritoneal carcinomatosis resistant to systemic treatment, PITAC has shown the ability to induce pathological regression and effectively control ascites [25]. Theoretically, PITAC achieves both pleurodesis and an antitumor effect by delivering high-pressure aerosolized chemotherapy into the thoracic cavity. It ensures even and homogeneous drug distribution across endothoracic surfaces, enabling direct treatment of pleural disease localizations that are difficult to reach with systemic therapy [24,25].

Given these encouraging findings and the limited therapeutic options for unresectable PM, we aimed to investigate PITAC as a treatment for MPE in this patient population.

## 2. Materials and Methods

We designed a pilot study to evaluate the safety and efficacy of PITAC in patients with unresectable PM presenting with MPE. Additional inclusion criteria were age between 18 and 80 years; Eastern Cooperative Oncology Group (ECOG) performance status ≤ 2, suitable for general anesthesia; life expectancy of at least three months; and liver, kidney, and cardiopulmonary function within 10% of the normal range. Previous treatments (e.g., surgery for other malignancies, chemotherapy, or hyperthermic intraperitoneal chemotherapy—HIPEC) were not deemed exclusion criteria; however, final eligibility was determined during multidisciplinary tumor board (MDTB) discussions, taking into account comorbidities, functional reserve, and tumor burden.

Given the preliminary and exploratory nature of this study, no control or comparison group was included. The aim was to evaluate safety, technical feasibility, and early efficacy results of the PITAC procedure.

The study was approved by the Institutional Review Board of the University Hospital of Pisa and registered in the Clinical Trial Information System (CTIS—EuCT No: 2024-511255-17-00). All procedures conformed to the principles of the Declaration of Helsinki. Written informed consent was obtained from each patient for the collection, analysis, and publication of prospectively gathered anonymized data.

### 2.1. PITAC Procedure

The patient is positioned in lateral decubitus under general anesthesia with double-lumen intubation. Two 12 mm balloon trocars (Applied Medical, Rancho Santa Margarita, CA, USA) were inserted into the chest wall: one in the seventh intercostal space (ICS) along the mid-axillary line, and the other in the fifth ICS along the anterior axillary line. Balloon trocars ensured a closed system and prevented aerosolized chemotherapy leakage from the thoracic cavity.

A standard thoracoscopy was then performed, with aspiration of MPE and lysis of any pleural adhesions. A safety checklist was followed to ensure maximal protection for both the patient and staff. An intrathoracic pressure of 12 mmHg was established using normothermic carbon dioxide (CO_2_), and a CE-certified, medical-grade stainless-steel nebulizer (Reger GmbH, Villingendorf, Germany) was inserted through a trocar and connected to a high-pressure injector.

All staff left the operating room (OR) during chemotherapy aerosolization to avoid exposure. Using a remote control, cisplatin (10.5 mg/m^2^ in 150 mL 0.9% NaCl) and doxorubicin (2.1 mg/m^2^ in 50 mL 0.9% NaCl) were sequentially aerosolized into the pleural cavity at a maximum pressure of 220 pound-force per square inch (PSI) and a flow rate of 0.7 mL/s, as specified by the nebulizer manufacturer. Vital signs and the aerosolization process were monitored remotely by both surgeons and anesthesiologists. Although laminar airflow systems minimize inhalation risk, remote monitoring was used as an additional safety measure.

The closed system remained in a steady state for 30 min at 37 °C under constant intrathoracic CO_2_ pressure (12 mmHg) to enhance drug penetration into neoplastic tissue. Afterward, the team re-entered the OR wearing protective barrier garments, including aerosol masks, protective eyewear, and double-layer gloves. The aerosolized chemotherapy was evacuated via a closed surgical smoke evacuation system equipped with dual micro-particle filters to capture residual molecules.

Finally, the trocars were removed, and two chest drains were positioned according to the standard protocol.

**Figure 1:**
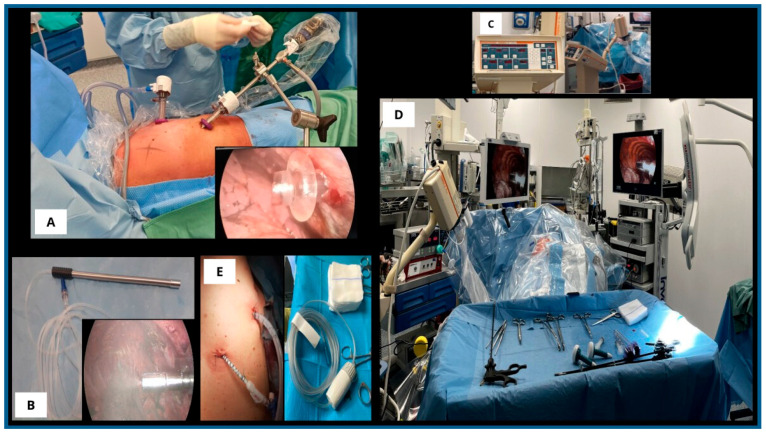
**PITAC procedure.** (**A**): Patient in lateral decubitus position with two 12 mm balloon trocars: one placed in the seventh ICS along the mid-axillary line and the other in the fifth ICS along the anterior axillary line; intraoperative view of a 12 mm balloon trocar. (**B**): CE-certified nebulizer during the intrapleural nebulization of chemotherapeutic agents. (**C**): High-pressure injector connected to the nebulizer for sequential aerosolization of cisplatin and doxorubicin into the pleural cavity. (**D**): Operating room setup during the procedure, remotely monitored from outside. (**E**): Surgical smoke evacuation system with dual micro-particle filters; final placement of tubular and pigtail chest drains.

The procedure is illustrated in Figure 1.

### 2.2. Chemotherapy Regimen

Chemotherapeutic agents were selected based on current literature recommendations [26]. Cytostatic solutions were prepared by trained pharmacists according to medical prescriptions, with dosages calculated using the patient’s body surface area (BSA) via the Boyd formula. The regimen included Cisplatin (Iketon, Milan, Italy) at 10.5 mg/m^2^ in 150 mL of 0.9% NaCl and Doxorubicin (Pharmacia, Milan, Italy) at 2.1 mg/m^2^ in 50 mL of 0.9% NaCl.

## 3. Results

From January 2022 to December 2024, nine patients with unresectable PM were enrolled in our study, and a total of eleven PITAC procedures were performed (two patients underwent a second PITAC). No intraoperative or postoperative complications occurred, and there was no 30-day mortality. The mean operative time was 121.6 ± 25.0 min. No OR contamination by aerosolized chemotherapeutic agents was detected at any time. The median chest tube duration was 2 days (IQR: 1), and the median hospital stay was 4 days (IQR: 1). No evidence of systemic toxicity or hypersensitivity reactions to the chemotherapeutic agents was observed during hospitalization or throughout the early and mid-term follow-up (FUP) periods. Patient characteristics are summarized in Table 1.

Among the cases, one patient exhibited particularly impressive and unexpected oncological outcomes. A 79-year-old man, previously treated for peritoneal MM (2018), was referred to our tertiary care university center in October 2021 for worsening dyspnea and right pleural effusion. His medical history included prior tobacco use, occupational exposure to asbestos, systemic hypertension, and a previous episode of pulmonary embolism and deep vein thrombosis, for which he was receiving anticoagulant therapy. In 2017, the patient was diagnosed with the omental involvement of epithelioid MM. In 2018, he received three cycles of neoadjuvant chemotherapy (CHT), followed by cytoreductive surgery, including total peritonectomy, splenectomy, cholecystectomy, appendectomy, and HIPEC with mitomycin. FUPs were conducted regularly until May 2020, when the recurrence of peritoneal MM was identified. The patient subsequently underwent five cycles of chemotherapy with carboplatin (CBDCA) and pemetrexed, followed by reoperation via laparotomy, including ileocolic resection, excision of parietal and visceral tumor nodules, and a second HIPEC procedure with cisplatin (CDDP) in October 2020. Postoperatively, adjuvant CHT was administered using a CBDCA + pemetrexed regimen for nine cycles. Maintenance therapy with pemetrexed every 21 days was initiated thereafter, in conjunction with regular FUPs using computed tomography (CT).

**Figure 2:**
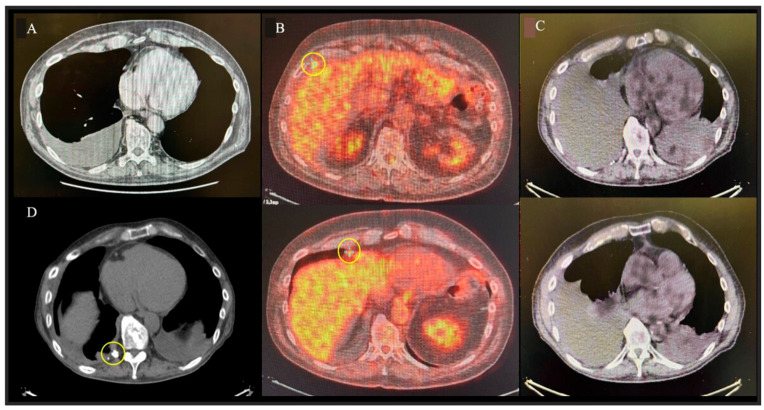
**Imaging findings.** (**A**): Chest CT before first PITAC showing right pleural effusion. (**B**): PET-CT before first PITAC showing two FDG-avid right pleural lesions (circled). (**C**): PET-CT before second PITAC showing effusion recurrence without FDG uptake. (**D**): Follow-up CT one year after second PITAC showing no effusion recurrence; biopsy site clips are circled.

In October 2021, the patient was referred for evaluation of progressive dyspnea. Chest ultrasonography revealed a significant right-sided pleural effusion, and a diagnostic and therapeutic thoracentesis was performed, yielding 700 mL of pleural fluid. Cytological analysis was consistent with a malignant mesothelial neoplasm. Chest CT scan confirmed an increase in the right pleural effusion without significant mediastinal or intra-abdominal disease (Figure 2A). Subsequent 18F-fluorodeoxyglucose positron emission tomography (18F-FDG PET) identified two hypermetabolic lesions located at the level of the right VI–VII intercostal space and in the right cardiophrenic angle (SUV max 6.2—Figure 2B). The case was reviewed by the MTDB, which deemed the patient eligible for PITAC. Thoracic cytoreductive surgery was instead excluded based on the patient’s prior oncological history, age, and comorbidities.

**Figure 3:**
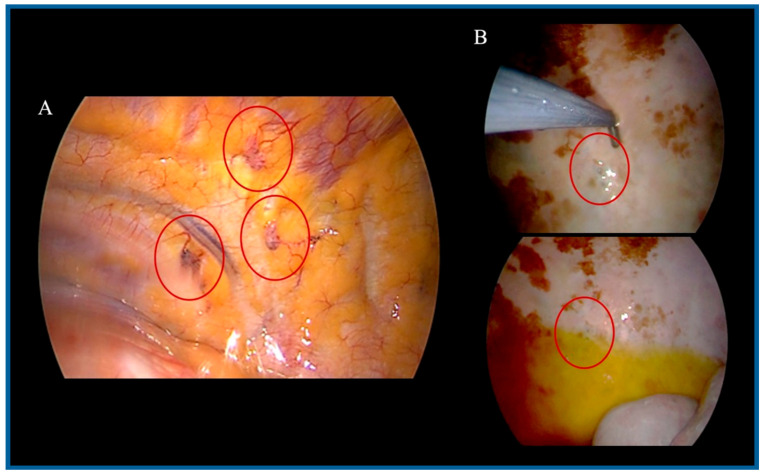
**Surgical views.** (**A**): First PITAC: pleural disease with visible lesions (circled). (**B**): Second PITAC: fibrotic areas and new biopsies; prior biopsy sites marked with titanium clips (circled).

**Figure 4:**
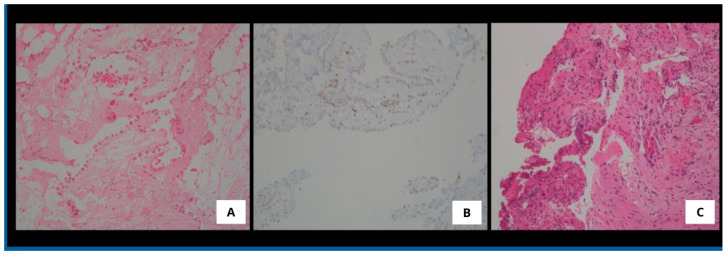
**Histopathological features.** (**A**): Parietal pleura showing a single layer of cuboidal mesothelial cells with mild cytological atypia (hematoxylin and eosin stain, 10× magnification). (**B**): Loss of BAP1 protein expression in tumoral mesothelial cells, with preserved staining in non-neoplastic inflammatory cells (immunohistochemical stain, 10× magnification). (**C**): Parietal pleura lacking mesothelial lining, with the presence of fibrous tissue and inflammatory cells only (hematoxylin and eosin stain, 10× magnification).

In February 2022, the patient underwent the first PITAC procedure without perioperative complications (Figure 3A). Titanium clips were placed at the biopsy sites during surgery to facilitate future localization. The postoperative course was uneventful, and the patient was discharged on postoperative day 4. Histopathological examination of pleural biopsies confirmed the diagnosis of mesothelioma in situ, with immunohistochemistry (IHC) positive for CKpan, calretinin, podoplanin, and WT-1, and negative for BAP1, Ber-EP4, and TTF-1 (Figure 4A,B). Due to underlying renal insufficiency, systemic CHT was not administered. PITAC was effective in achieving pleurodesis and local disease control for nine months.

In December 2022, follow-up 18F-FDG PET imaging showed recurrence of the right pleural effusion, although no pathological FDG uptake was observed (Figure 2C). A second thoracentesis was performed, but cytology was inconclusive for technical reasons. After reassessment by the MTDB, the patient was once again deemed eligible for PITAC.

In February 2023, he underwent a second PITAC procedure. Biopsies were obtained from previously marked sites using titanium clip localization, as well as from at least three additional pleural areas (Figure 3B). Histopathological examination revealed no evidence of malignancy (CKpan-, calretinin-, podoplanin-, WT-1-, EMA-, and CK5/6-), with findings consistent with chronic lymphoplasmacytic inflammation and fibrosis (Figure 4C). The intraoperative and postoperative course was uneventful, and the patient was discharged on postoperative day 4.

At the one-year FUP, CT imaging showed no recurrence of pleural effusion (Figure 2D).

## 4. Discussion

Given the encouraging results of pressurized intraperitoneal aerosol chemotherapy (PIPAC), which is well established and routinely used in the treatment of peritoneal carcinomatosis, including MM [25,26,27,28,29], we aimed to extend this innovative approach to patients with unresectable PM, in light of the scarcity of safe and effective alternatives for managing MPE. The principal advantage of PITAC lies in its ability to deliver aerosolized chemotherapeutic agents directly into the pleural cavity, leveraging the uniform distribution properties of gas within a closed space [24,25]. Pressurization of the pleural cavity via CO_2_ insufflation generates a pressure gradient between intrapleural and extrapleural compartments, enhancing the diffusion of fluids and drugs across the pleural surface. Additionally, this pressure may counteract the elevated interstitial fluid pressure typical of solid tumors, which is a major factor limiting drug absorption and contributing to chemoresistance [27]. By delivering chemotherapy in aerosol form under pressure, PITAC achieves homogeneous drug distribution throughout the pleural cavity. This permits the direct targeting of pleural metastases that are otherwise difficult to access via systemic chemotherapy [30]. The combination of aerosolization and CO_2_-induced pressure also improves tissue penetration, thereby permitting the use of lower chemotherapeutic doses compared to HITHOC methods. This, in turn, reduces systemic drug absorption and minimizes toxicity [31].

While primarily designed for symptom palliation in patients with pleural carcinosis, PITAC has the potential advantage of treating the underlying neoplasm as well as controlling the extent of MPE. We acknowledge that this proposed twofold mechanism is based on pharmacologic rationale and clinical observation. Cisplatin-based intracavitary chemotherapy, known for its cytotoxic rather than sclerosing properties, has been proven safe and effective in intrapleural surgery [15,29]. On the other hand, the use of chemotherapeutic agents with intrinsic sclerosing activity—such as anthracyclines (e.g., epirubicin and doxorubicin)—is aimed at inducing a diffuse intrapleural inflammatory response. This reaction promotes local activation of the coagulation cascade, leading to fibrin deposition and ultimately resulting in pleurodesis [32]. Therefore, PITAC combining the cytotoxic and sclerosing effect, could represent an innovative and pioneering treatment for pleural carcinosis, acting on the phenomena underlying the formation of MPE, namely the tumor-related production of vasoactive mediators and the pleural inflammatory reaction. No direct evaluation of inflammatory or fibrotic markers was performed in this pilot study, and further mechanistic studies are needed to confirm this dual effect.

Although limited to eleven procedures, our experience supports these preliminary hypotheses. We advocate for the use of platinum-based agents in combination with anthracyclines, given their dual cytotoxic and sclerosing properties. Notably, effective pleurodesis was achieved in all patients at the one-month FUP, with sustained results observed in seven cases, persisting for over six months following PITAC. Moreover, in our series, this novel procedure proved to be technically feasible and safe. No perioperative complications or mortality were observed, and the hospital stay was short and uneventful. The procedure was well tolerated by all patients, with no evidence of systemic toxicity. Additionally, occupational health and safety standards were fully upheld, with no contamination of the OR detected at any time.

Another notable outcome was the apparent tumor response to PITAC, as evidenced by both macroscopic and microscopic findings. Although data in the literature are still limited, Hansen et al. reported a patient with MPE secondary to HER2-positive ductal breast carcinoma who underwent three PITAC procedures. Notably, while malignant cells were present in the pleural fluid prior to the first PITAC, they were absent after the third treatment [33]. In analogy to the Peritoneal Regression Grading Score (PRGS), which is used to evaluate histological response following PIPAC [34], the authors proposed a four-tiered Thoracic Regression Grading Score (TRGS) for assessing pleural biopsies. TRGS-1 indicates a complete histological response, defined as the absence of residual tumor and the presence of only regressive features. TRGS-2 denotes a major response, where regressive changes predominate over viable tumor cells. TRGS-3 reflects a minor response, characterized by the predominance of tumor cells with some regressive features, while TRGS-4 represents no response, with viable tumor cells and no signs of regression. In a separate case involving a patient with rectal adenocarcinoma, the same authors reported histological improvement from TRGS-3 prior to the first PITAC to TRGS-1 before the second procedure [33].

These findings support the hypothesis that PITAC may contribute to pleural disease control. Nevertheless, given the anecdotal nature of the evidence from a single case, caution is warranted in interpreting oncological response until validated in larger cohorts. The absence of malignant cells in the previously positive site one year earlier—during the first PITAC procedure—as well as in several additional pleural areas, and in the absence of any intercurrent systemic oncologic treatment, provides a potential indication of the technique’s capacity to control local disease progression. However, additional data are currently lacking, and further studies are needed to confirm these preliminary observations.

In the context of mesothelioma, the role of BRCA1-associated protein 1 (BAP1) as both a diagnostic and prognostic marker is well established. BAP1 is a tumor suppressor gene frequently inactivated in PM through either somatic or germline alterations. Loss of BAP1 nuclear expression, as detected by IHC, is a hallmark of malignancy and has been associated with favorable prognostic features, including improved response to platinum-based chemotherapy and prolonged survival [35]. In our study, the patient who demonstrated a remarkable oncological response to PITAC (complete histological remission and durable pleurodesis after two procedures) exhibited BAP1 loss. While this may in part explain the favorable outcome, it is important to note that BAP1 status was assessed in a subset of patients only. Among the four additional patients tested, three also showed BAP1 loss but did not experience the same degree of histopathologic response or long-term effusion control. This variability suggests that BAP1 loss alone may not be sufficient to predict PITAC responsiveness, and other factors, such as tumor histology, immune microenvironment, or prior treatments, may contribute to the observed heterogeneity in outcomes [36]. On the other hand, the index case was diagnosed with mesothelioma in situ, a condition that cannot be established on hematoxylin and eosin staining alone. According to current WHO classification criteria, the diagnosis of mesothelioma in situ requires the demonstration of molecular alterations associated with malignancy [10,37]. Specifically, loss of BAP1 nuclear expression by IHC, or detection of CDKN2A (p16) homozygous deletion by fluorescence in situ hybridization (FISH) or by MTAP IHC, must be present to support this diagnosis. In pleural mesothelial proliferations, the sensitivity of BAP1 IHC ranges from 50% to 65%, with higher rates in the epithelioid subtype and lower in sarcomatoid variants. It is important to note, however, that while BAP1 loss is a common feature of MM, its absence does not exclude malignancy. Therefore, the assessment of BAP1 loss and/or CDKN2A homozygous deletion is most valuable when histologic features are equivocal. In cases where malignancy is unequivocal, such ancillary testing may not be required for diagnostic confirmation. Given these preliminary findings, further investigation into the predictive role of BAP1 and other molecular markers in the context of PITAC is warranted. Future studies should incorporate standardized molecular profiling to explore correlations between tumor biology and therapeutic response, which could ultimately guide patient selection and optimize the clinical utility of PITAC.

While our results provide valuable insights, they should be interpreted in light of the following limitations. First, this study includes a small number of patients from a single institution, limiting the generalizability of results. Second, the absence of a control group precludes direct comparisons with standard therapies. Third, long-term oncological outcomes were not assessed in this preliminary evaluation. Finally, while histological regression was qualitatively described, scoring systems like the TRGS remain exploratory and not yet validated for routine use in thoracic oncology.

As mentioned, we are currently enrolling patients in a prospective phase II study with a larger sample size, which is specifically designed to evaluate not only pleural effusion control but also overall survival, quality of life, and treatment tolerability. We hope that this ongoing work will provide more definitive evidence regarding the clinical utility and safety of PITAC in this population with very limited therapeutic options and poor prognosis.

## 5. Conclusions

PITAC may represent an innovative and promising approach for the management of pleural carcinomatosis by targeting both MPE formation and tumor progression. Its ability to combine cytotoxic and sclerosing effects offers a dual therapeutic benefit, potentially improving both symptom control and local oncological outcomes.

Our preliminary findings demonstrate the feasibility, safety, and potential efficacy of PITAC in patients with unresectable PM, with encouraging results in terms of pleurodesis success and disease control.

Prospective clinical trials are needed to validate PITAC as a pivotal loco-regional treatment strategy for unresectable PM and associated MPE.

## Figures and Tables

**Table 1:** Patients’ characteristics.

Characteristic	Patients (n)
**Gender**	
Male	8
Female	1
**Median Age** (years, IQR)	74 (8)
**ECOG score**	
1	5
2	4
**Comorbidities**	
Hypertensive heart disease	7
Heavy smoking	2
Diabetes	2
Cerebrovascular disease	4
Occupational exposure to asbestos	6
**Histology**	
Mesothelioma in situ	1
Epithelioid	4
Biphasic	4
Sarcomatoid	0
**BAP-1 status**	
Positive	1
Negative	4
Not tested	4
**Post-operative treatment**	
None	2
Pemetrexed + Cisplatin	3
Pemetrexed + Carboplatin	1
Nivolumab + ipilimumab	3
**Absence of MPE recurrence**	
at 1 month	9
at 3 months	7
at 6 months	7
**Re-PITAC**	2
1 patient (M, mesothelioma in situ) after one year *	
1 patient (F, biphasic) after 9 months *	

n, number; IQR, interquartile range; ECOG, Eastern Oncology Cooperative Group; PITAC, pressurized intra-thoracic aerosol chemotherapy; * BAP1 status: negative.

## Data Availability

The article’s underlying data will be shared on reasonable request to the corresponding author.
